# Losing Your Sense of Smell: It Can Be Worse than Imagined. Comment on Becht et al. Losing Your Sense of Smell: How Bad Is It?—A Comparative Study on the Personal Importance of Smell. *Brain Sci.* 2025, *15*, 218

**DOI:** 10.3390/brainsci15080867

**Published:** 2025-08-14

**Authors:** Alexander Wieck Fjaeldstad, Thomas Hummel

**Affiliations:** 1 Flavour Clinic, University Clinic for Flavour, Balance and Sleep, Department of Otorhinolaryngology, Regional Hospital Gødstrup, Hospitalsparken 15, 7400 Herning, Denmark; 2 Flavour Institute, Department of Clinical Medicine, Aarhus University, Palle Juul-Jensens Boulevard 99, 8200 Aarhus N, Denmark; 3 Smell & Taste Clinic, Department of Otorhinolaryngology, Faculty of Medicine Carl Gustav Carus, Technische Universität Dresden, Fetscherstraße 74, 01307 Dresden, Germany; thomas.hummel@tu-dresden.de

Becht et al.’s article “Losing Your Sense of Smell: How Bad Is It?—A Comparative Study on the Personal Importance of Smell” makes an important contribution by demonstrating that 92% of Dutch university students would relinquish olfaction before vision or hearing and that prior brief smell loss did not appreciably alter this view [1]. The study elegantly confirms the long-standing cultural tendency to rank smell last among the senses, which compares to previous work, e.g., Herz and Bajec [2]. Yet, by relying on an imagined sensory deficit in a largely normosmic population, it also reveals how poorly intact smellers grasp the everyday significance of olfaction.

Recent reviews show that smell loss remains “an invisible disability … hard to even conceive of” for laypeople and professionals alike [3]. Becht et al.’s findings do not surprise; they quantify the gap in awareness that underlies decades of under-diagnosis and under-funding of olfactory disorders. However, because imagination is bound by prior experience, surveys of healthy participants risk underestimating the consequences of genuine anosmia or hyposmia. The lived reality is revealed only when asking those who have permanently lost their sense of smell.

Patients born without olfaction report significantly more household accidents, greater social insecurity, and a higher prevalence of depressive symptoms than matched controls [4]. Those who acquire loss later in life describe diminished food enjoyment, impaired hazard detection, and reduced quality of life across multiple domains [5]. Long-COVID cohorts reinforce this picture: persistent post-viral olfactory dysfunction independently lowers the mental component of the SF-12, even six months after infection [6]. Collectively, these data show that real smell loss affects nutrition, safety, social bonding, and emotional well-being—outcomes that cannot be fully appreciated by individuals whose olfactory input remains intact.

Becht et al. nevertheless provide a valuable springboard for education. Their observation that students would trade smell for relatively trivial commodities highlights just how discordant public perception is with clinical reality. Becht et al. also uncovered notable sex-related effects. Women assigned a lower value to olfaction than men and were more willing to forgo it in exchange for commodities such as money or hair. This pattern contrasts sharply with the patient mix seen in chemosensory clinics, where women constitute the majority of individuals seeking help for smell disorders [7]. Another striking result was that participants who had experienced ≥2 weeks of prior olfactory dysfunction still ranked smell last and remained willing to relinquish it, contrary to the authors’ initial hypothesis. Future studies could disentangle this paradox by stratifying prior loss according to severity and permanence. A brief, fully reversible hyposmia may leave little lasting impression, whereas a participant who is still anosmic or coping with severe parosmia would view another “loss” as largely inconsequential—thereby masking the true burden of persistent olfactory impairment. Boesveldt & Parma remind us that millions live with chronic olfactory disorders, reporting “negative impact on mental and emotional health, increased social isolation, and associated financial difficulties” [3]. Therefore, bridging the perceptual gap requires studies that combine imaginative valuation tasks with testimonies and objective metrics from patients. Including participants with acquired anosmia, parosmia, or congenital anosmia would allow direct comparison between hypothetical and lived consequences, while controlling for duration and cause of dysfunction—variables the present work could not address.

In summary, Becht et al. convincingly document the low priority accorded to smell by healthy young adults, thereby underscoring the need for public-health messaging about olfaction’s role in safety, diet, and psychosocial health. Future research focusing on patient experience will clarify the full burden of smell loss and may motivate earlier diagnosis and therapeutic development. Until then, the current paper stands as a timely reminder that what people think they can live without often proves indispensable once it is gone.
